# Nutrition Recommendations for Table Tennis Players—A Narrative Review

**DOI:** 10.3390/nu15030775

**Published:** 2023-02-02

**Authors:** Liyan Huang, Jeremy W. C. Ng, Jason K. W. Lee

**Affiliations:** 1 Sport Science and Medicine Center, Singapore Sport Institute 3 Stadium Drive, Singapore 397630, Singapore; 2 High Performance Department, Singapore Table Tennis Association, 297-C Lor 6 Toa Payoh, Singapore 319389, Singapore; 3 Human Potential Translational Research Programme, Yong Loo Lin School of Medicine, National University of Singapore, Singapore 117593, Singapore

**Keywords:** racket sport, ping pong, indoor sport, diet, recovery

## Abstract

Table tennis (TT) is the second most popular racket sport globally and was the sixth most widely played Olympic sport in 2005. It is an indoor racket sport requiring a mixture of power, agility, alertness and fast reactions. Players need to move quickly around a table to receive the ball and produce powerful returns. New rules such as increased ball size and a change in ball material have changed the ball’s trajectory, increasing the overall duration and intensity of game play. Scientific research on TT is growing but there has been no systematic review of nutrition for the sport. This review provides nutritional recommendations for TT athletes based on the physiological demands of TT, including energy expenditure during training and competitions, and the main metabolic pathways of TT. Guidelines on the daily intakes of carbohydrate, protein and fat are discussed in addition to hydration strategies. Micronutrients of concern for TT athletes include iron, magnesium and vitamin D and their recommended intakes are also provided. The timing and dose of ergogenic aids that may improve TT performance such as caffeine, creatine, lutein and zeaxanthin and beta-alanine are reviewed. Specific nutritional strategies for intakes leading up to competitions, post training and competition recovery and nutritional strategies for travel are also addressed.

## 1. Introduction

Table tennis is an indoor racket sport that requires a mixture of power, endurance, agility, mental alertness and quick reactions. The rules of table tennis are similar to tennis except that the sport is played on a 2.7 m (9.0 ft) long, 1.5 m (5.0 ft) wide and 0.76 m (2.5 ft) tall table. The aim of each point is to send the ball to the opponent’s side of the table such that the opponent is unable to receive the ball or produce a valid return.

Although table tennis was first included as an Olympic sport in 1988, its history began in the 1880s after it was adapted from lawn tennis to a dining table with improvised equipment [1]. The game went through several iterations in the subsequent years and gained popularity from 1900 after celluloid balls were used [1]. The Table Tennis Association was formed in 1901 and the game was introduced in China through Western settlement and trade missions [1]. 

Table tennis debuted in the Olympic Games with four events (men’s and women’s singles and doubles) being played [2]. In 2008, the doubles events were replaced by team events, and in 2020, mixed doubles was introduced as an additional category [3]. Prior to 2001, games were won when a player (or pair of players for doubles) first reached 21 points. The International Table Tennis Federation (ITTF) subsequently adopted an 11-point scoring system to make game play faster and more exciting [4]. For both singles and doubles events, competition matches consist of the best of an odd number of games, typically five or seven games [1]. For team events, three players will play the best of five matches. An Olympic Games team match comprises a minimum of two singles matches and one doubles match with up to two additional singles matches being played until one team wins three matches in total [2]. World Championship team events, on the other hand, consist of best of five singles matches [5]. Team events are completed in a single day and each player plays a maximum of two matches. 

The current rules of table tennis specify that the game is played with a ball with a mass of 2.7 g and a diameter of 40 mm [5]. In 2000, the diameter of the ball was increased from 38 mm to 40 mm to reduce the speed of play of the sport, lengthen rallies and to make it more viable as a televised spectator sport [6]. Changing the ball dimensions had great implications on overall game play as the ball must overcome greater air resistance and resulted in reduced speed and longer rallies [6]. In addition, the material of the table tennis balls was changed from celluloid to plastic in 2014 due to safety concerns around the highly flammable celluloid material [7]. This change resulted in changes in the trajectory of the ball. Plastic balls with high speed and top spin accelerate more upon collision compared to celluloid balls, resulting in a greater intensity of game play [8]. 

Well-designed nutrition strategies can enhance sporting performance as well as recovery from sporting activities [9]. The timely intake of carbohydrate has been shown to improve endurance, while the co-ingestion of carbohydrate and protein after exercise can improve recovery [9]. However, there has been a paucity of table-tennis-specific nutrition research. Understanding the physiological, sporting and competition demands of table tennis can allow the application of existing sport nutrition knowledge to provide evidence-based recommendations for table tennis. 

Table tennis is the second most popular racket sport in the world and was the most watched sport in China for the 2021 Olympic Games, registering close to 350 million unique viewers [10,11]. A recent restructure in the ITTF saw the launch of World Table Tennis (WTT) in 2021, along with organization of the Grand Smash event, offering the highest ever prize pool of USD 2 million [12]. 

To the best of the authors’ knowledge, there has not been a formal review on the nutrition requirements of table tennis. With the growing popularity and commercial value of table tennis, a review is timely. The aim of this review is to evaluate the current scientific literature on the physiological demands of table tennis and provide nutritional recommendations including energy, carbohydrate, protein and selected micronutrients, along with potential research areas for future work. 

## 2. Methodology

A narrative review was conducted between January 2021 to June 2022. Peer-reviewed journal articles in the English language were located through searches from PubMed and Google Scholar from inception up to June 2022. The terms used for the search included table tennis and physiology or metabolism to identify relevant literature on the physiological requirements of table tennis. Another search was conducted using the search terms table tennis and nutrition or body composition to identify relevant articles on this specific population of athletes. Other relevant references related to the topic of discussion (e.g., the nutrition management of jet lag) were then manually searched and identified.

## 3. Results and Discussion 

### 3.1. Physiological Challenges of Table Tennis 

#### 3.1.1. Optimum Physique 

Body mass and composition are known factors that can influence sports performance [13]. There is no optimal body type or shape for table tennis due to the limited data available [14]. Height and body mass data of the top 100 male players found no correlation between performance and anthropometric measurements [15]. Studies conducted among competitive Brazilian and Chilean table tennis also did not find any relationship between performance and skinfold measurements as well as fat-free mass [16,17]. 

Anthropometry data of table tennis players from traditional powerhouses such as China and Japan are currently lacking. As table tennis requires agility and power, leanness and a high power to weight ratio will likely benefit athlete performance. Tracking body composition allows sport scientists to monitor the efficacy of training programs and nutrition interventions and work towards the optimal composition for the individual athlete [13]. 

#### 3.1.2. Training and Competition Demands of Table Tennis 

Competitive table tennis is governed by the ITTF and they sanction global events such as the ITTF World Tour and Challenge Series as well as the WTT series of events. As competition events are held throughout the year, table tennis players spend much of their time traveling for these events, as well as the World Cup, World Championships, major games (e.g., Olympic Games, Asian Games) and training camps. Prior to 2020, the Japanese table tennis team reported almost ten international trips for competitions annually [18]. World tour competitions typically last for seven days and high-ranked players are likely to play many games throughout. Besides being aerobically fit, timely fueling strategies and effective nutrition recovery practices are critical in maintaining high levels of performance throughout the year. 

Analyses of matches at the 2004 Olympic Games in Athens and 2008 Olympic Games in Beijing showed that table tennis game play times ranged from 3.7 min to 7.5 min [6,19]. The mean duration of the games increased as the competition progressed from the preliminary rounds to the finals. The game play times for men ranged from 3.8 min to 5.5 min, while for women, it ranged from 3.7 min to 7.5 min [6,19]. The average men’s match duration in the Beijing Olympic Games was 27.5 min and the women’s duration was 32.3 min [6]. However, when the ball size increased from 38 mm to 40 mm, the average rally time per point increased by up to 10 s [4]. Although match play times are short compared to other racket sport such as tennis, table tennis competitions can be as physically demanding as other racket sports, and players may also need to play multiple matches in a day (i.e., for singles and doubles or for team events) with competitions typically lasting for one week [20]. Such competition structures further emphasize the need for timely and appropriate refueling for optimal physical performance on multi-match days. 

**Figure 1:**
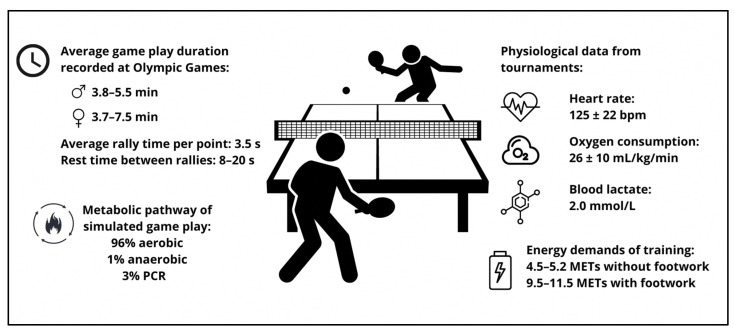
Summary of the physiological demands of table tennis game play and training determined through data collected from official Games and tournaments and simulated game play from research studies.

Data obtained from official tournaments of regionally and internationally experienced Brazilian table tennis players reported blood lactate values, heart rate and oxygen consumption of 2.0 mmol·L^−1^, 125 ± 22 bpm and 26 ± 10 mL·kg^−1^·min^−1^, respectively [21]. These values are similar to those reported for simulated badminton and tennis match play [22,23]. A review by Zagatto et al. (2017) found that the duration of a table tennis rally averages 3.5 s for both elite and sub-elite players, while rest time ranges from 8 to 20 s depending on tournament structure. The resulting effort–rest ratio is 0.12 to 0.5 [14]. The metabolic pathways of simulated table tennis game play showed that 97% (±2%) were aerobic in nature, 1% (±0.7%) anaerobic and 3% (±1%) phosphocreatine breakdown [24]. Despite the small contribution of the anaerobic pathway, simulated table tennis game play showed that greater anaerobic capacity enabled rallies to be played at a higher intensity [25]. Considering the nature of table tennis game play, this suggests that having greater anaerobic capacity may be advantageous in allowing more explosive strokes and returns. Intake wise, these parameters suggest that carbohydrate should be the main source of fuel for table tennis players, while ensuring sufficient creatine intake to saturate the muscle storage of phosphocreatine. The relatively short rally duration also suggests that nutrients that enhance mental sharpness and quick reaction times may be useful. Future research can consider other forms of physiological and strength profiling that have greater sensitivity in profiling the physiological requirements of table tennis. The physiological demands of table tennis are summarized in Figure 1. 

**Figure 2:**
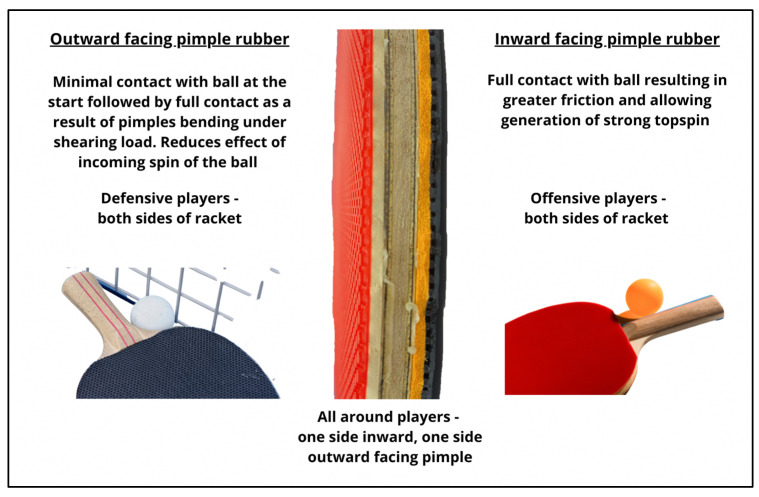
Commonly used rubber coverings on table tennis rackets and their impact on table tennis playing styles.

There are three main playing styles in table tennis and they comprise defensive, offensive and all-around play [25]. Offensive play is fast-paced and typically involves technical and tactical manipulation to score a winner (i.e., a rally ends with the ball bouncing off the opponent’s side of the table without being hit by the opponent), while all-round play is usually less aggressive and aims to force an error from the opponent [25]. Most offensive players tend to impart topspin to the ball, while all-round players impart topspin to the ball during attacks and underspin to the ball to defend [26]. Players with different playing styles use different types of rubber coverings for their rackets, either inward or outward facing pimples. Defensive players usually have both sides of their rackets covered with outward pimple rubber coverings. Offensive players, on the other hand, usually equip inward pimple rubber coverings on both sides, while all-round players have an inward pimple rubber covering and an outward pimple rubber covering on the other side. There are over 1600 rubber coverings approved by the ITTF as of 2019 [27], with variations in the height of the pimples, rubber layer and the concentration of the pimples. A pictorial representation of the rubber coverings and associated playing styles is shown in Figure 2.

Varenberg & Varenberg [28] investigated the influence of rubber coverings on the ball and found that an inward pimple rubber covering possesses the highest friction force due to full contact of the rubber and ball. This allows a player to produce strong topspin favoring an offensive playing style [29]. Unlike the inwards pimple covering, the ball comes into minimal contact with the outwards pimple covering at the beginning, followed by full contact when the pimples bend under shear load [28]. This minimizes the effect of incoming ball spin and allows the player to gain control of the ball by adding a spin reversal. Anecdotal evidence from players suggests that when offensive players play against each other, one can re-direct the force of the oncoming ball back to the opponent without generating much from their own [30]. However, when an offensive player plays against a defensive orientated player, the offensive player needs to first counter the oncoming underspin of the ball with topspin. The oncoming ball with under-spin is also usually slow in speed. The offensive player is first required to overcome the underspin by applying top spin and subsequently generate forward power to the ball to increase the speed of the ball back to the opponent. This not only increases the physical demands on the offensive player, it also requires the offensive player to have sufficient muscle strength to perform both actions for a forceful return. Ensuring sufficient protein intake is hence necessary for table tennis players. 

Although not different in terms of physiological parameters, a study comparing offensive and all-around playing styles showed significant differences in the rate of shots per rally [25]. The authors also found strong negative correlations between energy contribution from the phosphocreatine pathway and the number of shots per rally for offensive players while strong correlations were found between energy contributions from the oxidative pathway and VO_2_max with rally duration and the number of shots per rally for all-round players. Only all-round players presented strong significant correlations of energy contribution from the oxidative pathway and VO_2_max with rally duration and the number of shots per rally [25]. The authors suggested that all-round players challenge the phosphocreatine energy system to a lesser extent and rely slightly more on the oxidative system [25]. Another study found that compared with playing against offensive players, playing against defensive players usually results in a longer duration of rallies (5.4 ± 0.7 s vs. 3.2 ± 0.4 s), longer real playing time (453 ± 100 s vs. 235 ± 44 s) and consequently a higher mean heart rate (146 ± 6 bpm vs. 140 ± 9 bpm) and rating of perceived exertion (RPE) [31]. These results suggest different playing styles may utilize different energy systems to achieve better performance and this can be assisted with nutrition.

The training hours of elite table tennis vary according to country and teams although training typically consists of both ball training and strength and conditioning training sessions [20]. For example, elite Dutch table tennis players train on average 14.5 ± 6.2 h per week [32], while the Singapore national table tennis players train up to 26 h per week [33]. Anecdotal reports from players training with the Chinese provincial team suggest that the weekly training duration ranges from 30 to 35 h [30]. The average heart rate of table tennis players during training was found to be 142 beats·min^−1^, lower than the average heart rate during competitions which ranged from 162 to 172 beats·min^−1^ [34]. However, heart rates during training are heavily dependent on the elements of the training program and more demanding training will yield heart rates in excess of those measured in competitions [20]. 

#### 3.1.3. Energy Expenditure

The energy expenditure of table tennis players is largely dependent on their training load and intensity as well as individual characteristics such as body weight and composition [35]. Determined using doubly labelled water, male Japanese collegiate table tennis players training three hours daily had an estimated daily energy expenditure of 3700 ± 450 kcal [36]. In the same study, the authors found that the metabolic demands of table tennis practices ranged from 4.5–5.2 metabolic equivalents (METs) without footwork to 9.5–11.5 METs with footwork [36]. These findings were similar to Shieh et al. (2010) [37] who demonstrated that table tennis training and simulated match play had metabolic equivalents of 8.5 METs and 10.5 METs, respectively.

Existing data suggest that table tennis is a moderate intensity sport, and that tailoring energy requirements to intake can help athletes manage their physique and optimize their intake to suit the aerobic nature of training and competition. Assessing the nutritional adequacy of elite table tennis player diets is a worthy area of future research.

### 3.2. Nutrition Recommendations for Table Tennis Players 

#### 3.2.1. Carbohydrate

**Figure 3:**
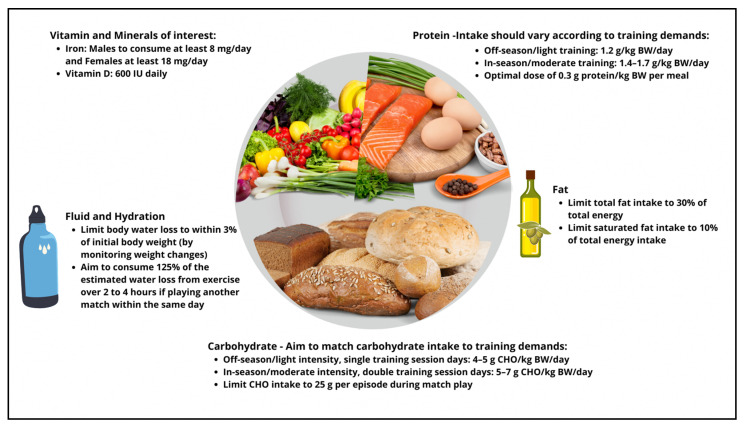
Summary of nutritional recommendations (macronutrients, key micro-nutrients of interest and hydration) for table tennis players based on the physiological demands of table tennis game play and training.

The main role of carbohydrate is to provide fuel for the muscles, especially during high-intensity exercises. Table tennis players’ daily intake of carbohydrate should be periodized to match the fuel needs of training and competition, especially on days with high training intensity or when it is important for athletes to train hard [38]. Guidelines on the daily intake of carbohydrate should be based on body mass and exercise load. Given the dominantly aerobic but moderate intensity of table tennis training and competition, carbohydrate recommendations for table tennis players in-season range from 5 to 7 g·kg^−^^1^ of body weight per day, depending on gender, duration and intensity of exercise as well as environmental conditions [38]. Intakes of carbohydrate should vary according to macro- and micro-cycles as described in Figure 3. 

Table tennis players should also consider the types of carbohydrate ingested. As it is a sport that relies heavily on cognitive function, mental alertness and speed of reactions, players should aim to maintain blood glucose levels before and during competitions by having appropriately timed meals and snacks. Players may benefit from consuming low to moderate glycemic index (GI) foods and meals before competition for sustained fuel release [39]. Refer to Table 1 for practical suggestions and examples of foods to consume.

#### 3.2.2. Protein

The Joint Position Statement on Nutrition and Athletic Performance [9] suggests that dietary protein intake ranging from 1.2 to 2.0 g·kg^−^^1^ of body weight per day is necessary to support metabolic adaptation, muscle repair, re-modeling and protein turnover. Training and table tennis game play require intermittent sprints involving powerful bursts of movement around the table. Hence, table tennis players should aim for a daily protein intake ranging from 1.4 to 1.7 g·kg^−^^1^ of body weight per day during pre-season and in-season, similar to the protein requirements of football and power sport athletes [40]. During the off-season, the training load of table tennis players is low and recommended protein intakes should be reduced to 1.2 g·kg^−^^1^ of body weight per day. 

Protein intake should be spread throughout the day for maximal absorption [41]. Athletes should aim to consume the optimal protein dose of 0.3 g·kg^−^^1^ of body weight after key exercise sessions and every three to five hours over multiple meals [9]. Overall daily energy intake must also be adequate to meet the energy expenditure needed to favor muscle protein synthesis [41]. High-quality protein such as whole milk, lean meat, soy and egg are preferred sources and their intake has been reported to increase muscle protein synthesis and protein accretion post exercise [9]. In situations where it is logistically difficult to consume protein foods or drinks (e.g., multiple-match days), protein powders may be consumed instead [42]. 

#### 3.2.3. Fat

Fat is an important component of an athlete’s diet as it provides energy, essential fatty acids and acts as a vehicle for fat-soluble vitamins to enter the body. The World Health Organization recommends that total fat intake be limited to 30% of the total energy intake, of which less than 10% is from saturated fat [43]. Fat intake for table tennis athletes should be in accordance with public health guidelines and individualized based on training level and body composition goals [9]. It is not recommended that table tennis players chronically consume a fat intake of below 20% of their total energy intake as it is likely to reduce the intake of essential fat-soluble vitamins and fatty acids [9]. Meals or snacks consumed immediately prior to training or competition should be lower in fat to minimize any gastrointestinal discomfort [9]. 

#### 3.2.4. Fluid and Hydration

It is important to maintain optimal hydration status during exercise as hypo-hydration can adversely impact exercise performance and increase physiological strain, especially in hot environments [9] (refer to Figure 3 for guidelines and Table 1 for strategies to obtain an optimal hydration status). A body water deficit in excess of two to three percent body mass has been shown in a laboratory setting to decrease exercise capacity by up to 30% [44]. The same level of dehydration can also negatively affect athletes’ ability to execute sport-specific skills [44]. More recently, Benton et al. (2016) [45] found that even a one percent loss of body mass due to hypo-hydration can adversely affect attention and cognitive function. Hence, promoting an ideal hydration status at practice is beneficial for optimal table tennis performance. As table tennis relies heavily on skill execution, alertness and reaction time, it is important that athletes begin training or competition in a well-hydrated state. Power output and endurance performance were impaired by up to three percent among athletes who were three percent dehydrated prior to exercise [46]. Athletes should also consume fluids after exercise to replenish the fluid and sodium lost during exercise. It should be cautioned that athletes should not consume fluids in excess to avoid hyper-hydration and weight gain. 

The types of fluids to consume are dependent on exercise duration and intensity. As a general guideline, athletes participating in high-intensity exercise for longer than one hour should consume a carbohydrate–electrolyte drink containing carbohydrate for additional fuel and electrolytes to help replenish those lost in sweat [38,46]. On competition days, when players must compete in multiple events and/or matches, consuming carbohydrate–electrolyte drinks during breaks and after matches is recommended for more efficient rehydration and fuel replenishment. For short-duration or low-intensity matches or training, water or electrolyte water should suffice [38]. During post-exercise recovery, players should aim to consume fluid at 125% of their estimated sweat loss (measured by changes in body weight) over two to four hours if they have another match within the same day [47].

Players should develop their individual hydration plans with a sport dietitian to follow before, during and after exercise to ensure that they are optimally hydrated. Players can monitor their sweat losses by weighing themselves before and after physical activity and develop their fluid intake regimes based on their individual sweat rates. 

#### 3.2.5. Key Micronutrient Requirements

Vitamins and minerals are essential in energy metabolism, hemoglobin synthesis, promoting good bone health and immune function as well as protecting the body against oxidative damage [9]. As physical activity increases energy expenditure, regular exercise may increase the turnover of B-group vitamins [48]. An increased sweat rate because of regular exercise also increases losses of minerals such as magnesium and zinc from the body [48]. As a result, athletes may require greater intakes of micronutrients, especially those who train for long durations or at high intensities with high energy expenditure. 

Table tennis players should aim to consume a wide variety of foods, especially fruits and vegetables, to ensure that they meet their requirements for all micronutrients. Although B-group vitamins are important for energy metabolism, deficiency is unlikely as staple foods such as bread and rice are often fortified with these vitamins [49]. Hence, micronutrients of greater concern for table tennis players are iron, magnesium and vitamin D. 

##### Iron

Iron is required for energy production and the formation of oxygen-carrying compounds, hemoglobin and myoglobin, and a deficiency in iron may compromise the body’s oxygen-carrying capacity and negatively affect endurance and immune function in athletes [9]. Iron deficiency is one of the most observed deficiencies among athletes and is more prevalent among female athletes, vegetarians and regular blood donors [9]. Thus, table tennis athletes who are female, vegetarian or regular blood donors should be regularly screened to assess and monitor their iron status. Table tennis players should aim to consume iron at a level slightly above the recommended daily allowance (RDA). The RDAs for iron for males and females are 8 mg and 18 mg, respectively [50]. 

##### Magnesium 

Magnesium is a cofactor for many enzymatic reactions and plays an important role in anaerobic and aerobic energy generation [51]. Magnesium also functions to regulate the cellular movement of calcium which is essential in skeletal and smooth muscle contraction [51]. An increased dietary intake of magnesium appears to have beneficial effects on exercise performance in magnesium-deficient individuals, but not in those with an adequate magnesium status [52,53]. There is increasing evidence to suggest that athletes do not consume enough magnesium from their diets [53,54]. Table tennis athletes should aim to consume magnesium according to the RDA which are 400–420 mg per day for males and 310–320 mg for females, respectively [51].

##### Vitamin D 

While vitamin D’s importance in maintaining good bone density and calcium regulation has long been established, emerging research suggests that vitamin D also plays a vital role in regulating skeletal muscle function and modulating immune and inflammatory responses [55]. 

The vitamin D status of athletes may affect physical performance. Ward and colleagues (2009) [56] found that vitamin D status was correlated with jump height, velocity and power in young post-menarchal teenage girls. Professional soccer players from the United Kingdom with low serum vitamin D concentrations also showed significant improvements in 10 m sprint times and vertical jumps, but not in their 1-RM (repetition maximum) bench and squat tests following eight weeks of 5000 IU per day of vitamin D3 supplementation [57].

Reports globally indicate that vitamin D insufficiency is widespread, including in countries with sunny climates [58]. Studies conducted on athletes found that the prevalence of insufficiency varies by sport, training location and skin color [55]. The prevalence appears higher in athletes who participate in indoor sports such as gymnastics and basketball, even if they reside in a sunny country such as Israel [59]. Deficiency among the general population in South Asian countries such as China and Korea are prevalent, at approximately 70% [60]. 

The principal source of vitamin D in the human body is via synthesis in the skin when exposed to ultraviolet B radiation [61]. However, skin production of vitamin D is variable and dependent on the degree of skin pigmentation, age, sunscreen use, clothing cover, latitude, and length and time of the day of the sun exposure [61]. As table tennis is an indoor sport and players train primarily indoors, they are at risk of a poor vitamin D status if they do not consume foods fortified with vitamin D or actively seek sun exposure. Global data on the prevalence of vitamin D insufficiency and deficiency among table tennis players are currently lacking. Given the importance of vitamin D to health and muscle function, table tennis players should consider having periodic blood tests to assess their vitamin D status and correct any deficiency or insufficiency should they arise. Low vitamin D levels can be corrected by consuming an oral supplement under medical supervision or by ensuring daily sunlight exposure. Readers may refer to the dosing strategies suggested by Bleizgys (2021) [62] for vitamin D supplementation. 

Based on the physiological demands of training and competitions, a summary of table tennis players’ carbohydrate, protein, fluid and micronutrient requirements are summarized in Figure 3 and strategies to achieve them are summarized in Table 1. 

**Table 1:** Practical strategies to achieve nutritional recommendations for table tennis athletes.

Nutrient	Practical Strategies to Achieve Recommended Intake
Carbohydrate	Aim for low–moderate GI carbohydrate foods where possible. Most whole fruits such as apples, bananas, berries, etc., have low to moderate GI [63]. Ensure that carbohydrate foods (e.g., rice, noodles, pasta, bread, etc.) are consumed at all meals and snacks. During in-season/moderate training periods, consume carbohydrate–electrolyte drinks during training to maintain blood glucose level during training. Consume foods containing both carbohydrate and protein (e.g., milk) to optimize post-exercise recovery.
Protein	Intakes of protein should be spread throughout day, between three main meals and two snacks. Recommended sources include milk, lean meats, soy products and egg. Consume foods containing both carbohydrate and protein to optimize post-exercise recovery. Optimal dose of protein per intake is 0.3 g·kg−1 of body weight. Consume slow-release protein (e.g., casein) at night before sleep to help improve muscle synthesis and recovery. Pre-bedtime protein dose should range between 25 and 40 g.
Fat	Choose low-fat foods where possible and limit intakes of foods high in saturated fat such as animal skin and fat. Polyunsaturated fat (including omega-3 and 6 fatty acids) is preferred and can be obtained from sources such as fish, seeds and nuts.
Fluid and Hydration	Ensure to begin exercise in a well hydrated state. Hydrate with carbohydrate–electrolyte drink during hard training sessions and when competing in multiple matches in a day. Hydrate with electrolyte drink during light training sessions.
Vitamins and minerals	Iron: Consume red meats at least twice weekly; for vegetarians, consume dark leafy greens with citrus foods for enhanced absorption. Magnesium Regular consumption of dark leafy green vegetables, whole grains, nuts and seeds. Vitamin D: Regular vitamin D monitoring and oral supplementation as required. Consume vitamin-D-rich foods such as fatty fish (e.g., salmon, tuna), eggs and mushrooms. Regular sunlight exposure

#### 3.2.6. Nutritional Ergogenic Aids

Nutritional ergogenic aids refer to substances that can confer athletes with a competitive advantage typically through enhancing energy metabolism and optimizing body composition [64]. Caffeine and creatine are ergogenic aids that may improve the performance of table tennis players during competitions, while emerging evidence suggests that lutein and zeaxanthin may benefit the performance of table tennis players. The following section discusses the nature of each ergogenic aid and their relevance to table tennis. 

##### Caffeine

Caffeine, a natural component found in certain plants is commonly ingested in the form of coffee, tea, cola drinks and energy drinks [65]. It has been shown to enhance several different forms of exercise performance including endurance, high-intensity team sport, strength-power performance and mental vigilance [65]. Caffeine appears to exert its ergogenic effects as an adenosine receptor antagonist and at the central nervous system level by attenuating the effects of central fatigue [65]. Hence, caffeine may have an important role in exercises where concentration, reaction times and technical/tactical skills have a major influence on both physical and mental performance [66].

Caffeine may be useful for table tennis as it is heavily reliant on quick reaction times and speed. During competitions, players are expected to play multiple matches a day especially if they take part in several events. Caffeine has also been shown to improve performance over consecutive days of simulated table tennis competition [67]. As table tennis competitions last for several days, players may consider consuming caffeine to improve and or maintain performance towards the end of their competition week. Caffeine ingestion can also be used to mitigate the negative effects of jet lag which will be discussed later. 

The safe and effective dose for the intake of caffeine ranges from 3.0 to 4.0 mg·kg^−1^ of body mass, as recommended by the European Food Safety Authority to improve physical performance and reduce the rate of perceived exertion during exercise [68]. Ideally, athletes should consume the caffeine product 1 h prior to exercise. Athletes should also consume caffeine in its anhydrous form rather than through coffee or tea as their caffeine content is highly variable and dependent on many factors such as the brewing and grinding of coffee beans [69]. In addition, there are also suggestions that other compounds in coffee and tea may cause the caffeine within it to be less effective than when consumed in the anhydrous form [65]. It should be noted that individual responses to caffeine vary and athletes should trial and determine their individual optimum dose prior to competition [70]. 

##### Creatine

Creatine is a compound derived from the amino acids methionine, glycine and arginine and is found predominantly in skeletal muscles [71]. The majority of skeletal muscle creatine exists in the form phosphocreatine (PCr) which provides a rapid but brief source of phosphate for the regeneration of adenosine triphosphate (ATP), an energy source during maximal exercise [72]. Supplementation increases the intramuscular stores of PCr, allowing for an increased capacity to perform high-intensity exercise as well as improving the rate of PCr resynthesis during recovery [72]. Guidelines on safe and effective doses of supplemental creatine to increase the body’s supply of creatine are summarized in Table 2.

Creatine supplementation has been shown to delay the onset of fatigue for repeated sprint performances [73], increase lean muscle mass [74], improve maximal power output among trained athletes [75] and improve post-exercise recovery [74]. Given that the phosphocreatine pathway is one of the metabolic pathways of table tennis game play and that the profile of table tennis game play involves players exerting maximal effort for each point repeatedly, creatine supplementation has the potential to delay fatigue during game play through the enhanced resynthesis of PCr. Creatine supplementation appears to have the potential to mitigate declines in skill execution following sleep deprivation [76]. Table tennis players may benefit from an acute dose of creatine supplementation prior to competitions if they experienced insufficient sleep when they travel or due to anxiety from competition demands. 

**Table 2:** Nutritional ergogenic aids of benefit to table tennis—Evidence and recommendations for intake.

Nutritional Ergogenic Aid	Relevant Studies Showing Improved Exercise Performance	Recommendation for Intake
Caffeine	300 mg (equivalent to about 4.0 mg·kg^−1^ of body mass) of caffeine consumed during 2.5 h of exhaustive exercise improved both complex cognitive function and physical performance post exercise [66]. Caffeine intake at 3.0 and 4.5 mg·kg^−1^ of body mass for two consecutive days improved exercise performances in a 10 min all-out exercise by 4% on the first day and 5% on the second day [67].	For mental performance and physical performance [68]: 3.0–4.0 mg·kg^−1^ of body weight 60 min prior to exercise Caffeine can be taken on consecutive days for consecutive-day competitions. Trial effects of caffeine prior to competition to determine individual’s optimal dose and dosing strategy.
Creatine	Supplementation to increase muscle creatine stores can enhance the rapid regeneration of ATP and delay the onset of fatigue for repeated sprint performances lasting 6 to 30 s and with short recovery periods of 30 s to 2 min [73,74].	Loading protocol [72]: (a) 0.3 g·kg^−1^ body weight·day^−1^ for 3–5 days or 20 g·day^−1^ of creatine for 5–7 days; followed by 0.03 g·kg^−1^ body weight·day^−1^ or 2–5 g·day^−1^ of creatine for maintenance (b) 3–5 g·day^−1^ of creatine for 4 weeks
Lutein and Zeaxanthin	Supplementation with 20 mg·day^−1^ of zeaxanthin or a mixed formula containing 26 mg·day^−1^ of zeaxanthin, 8 mg·day^−1^ of lutein and 190 mg·day^−1^ of omega-3 fatty acids for four months reduced visual motor reaction time by about 10% in young, healthy adults [77,78].	Goji berry or wolfberry is a rich source of zeaxanthin. Consume 15 g daily to obtain 20 mg of zeaxanthin. Green vegetables such as spinach, kale, pea, parsley and basil are rich sources of lutein. Consume a minimum of 100 g of green vegetables daily to obtain 8 mg of lutein.
Beta-alanine	Supplementation with 6.4 g per day of beta-alanine for 4 weeks improved performance in repeated sprint ability [79].	Consume products that are ‘slow-release’ to minimize any side effects [80].

##### Lutein and Zeaxanthin

Lutein and zeaxanthin are carotenoids that make up the macular pigment of the human eye and are also found in post-receptoral visual pathways [81]. These carotenoids protect the eye macular from damage from blue light, improve visual acuity and scavenge harmful reactive oxygen species [81]. Lutein and zeaxanthin improve visual performance by increasing the speed by which visual information is processed [77]. It is important for table tennis players to have quick visual reaction speeds as the distance between opponents is relatively short (2.7 m apart based on length of table) resulting in very fast-paced game play. Supplementation with lutein (8 mg/day) and zeaxanthin (20 mg/day) through natural foods such as goji berries and green vegetables for four months may increase visual processing speed, potentially allowing players to react more quickly to the oncoming ball and potentially increasing the rate of successful returns [78]. Although the benefits of lutein and zeaxanthin supplementation appear promising for table tennis, there is currently no performance data available and future research should be conducted to quantify the efficacy of these nutrients via supplementation. Recommendations on foods to consume to achieve 20 mg of zeaxanthin and 8 mg of lutein are presented in Table 2. 

##### Beta-Alanine

Beta-alanine, a non-proteogenic amino acid, is a precursor of carnosine synthesis [80]. The ingestion of beta-alanine has been consistently shown to increase muscle levels of carnosine, an intracellular buffer [80]. High-intensity exercises, especially exercises that involve the anaerobic metabolic pathways, result in the production and accumulation of lactic acid [82]. Beta-alanine supplementation can improve the performance of high-intensity exercises and delay fatigue through buffering exercise-induced acidosis [80]. Although the anaerobic metabolic pathway is only a minor energy contributor of simulated table tennis game play, and consequently resulted in low lactate production, most of the studies determining the physiological demands of table tennis were conducted prior to 2014, when the ball material was changed [21,24]. Furthermore, simulated table tennis game play showed that greater anaerobic capacity enabled rallies to be played at a higher intensity [25]. Together, these suggest that beta-alanine has the potential to enhance table tennis performance by allowing rallies to be played at a high intensity for a longer time. Future studies can also profile lactic acid production and accumulation during table tennis game play through simulated game play, with multiple matches in a day, similar to modern competition formats. 

Summary of the evidence promoting the use of the ergogenic aids and practical suggestions for their uses are summarized in Table 2. 

### 3.3. Specific Nutrition Strategies 

#### 3.3.1. Nutritional Management of Travel and Jet Lag 

International travel may pose a problem for athletes as travel stress and physiological disturbances resulting from crossing time zones may interfere with training and preparation for competition and transiently increase the risk of injuries [83]. To minimize disruptions to the circadian rhythm, intakes of protein and carbohydrate may be manipulated at mealtimes. A protein-rich breakfast increases plasma tyrosine levels and its uptake into the brain which in turn stimulates the synthesis and release of dopamine and norepinephrine [84]. This activates the body’s arousal system allowing the athletes to feel more alert [84]. A carbohydrate-rich meal in the evening on the other hand raises plasma tryptophan levels resulting in a higher tryptophan/tyrosine ratio and promotes the synthesis and release of serotonin [85]. As serotonin is the precursor of the sleep-inducing hormone melatonin, a carbohydrate-rich evening meal with a high glycemic index will allow the athlete to feel drowsy and inclined to sleep [85]. 

Melatonin may also be consumed to induce drowsiness and sleep [83]. It has also been shown to reduce the subjective symptoms of jet lag after real or simulated flights and improved sleep in a laboratory environment [83]. On the other hand, to promote wakefulness and the resynchronization of the circadian system following long-haul travel, the ingestion of caffeine in the mornings after arrival may be beneficial [86].

#### 3.3.2. Nutritional Strategies for Competition 

In the days leading up to competition, table tennis players should consume carbohydrate-rich meals according to the daily guidelines in Figure 3 to ensure that their glycogen stores are replete. On competition days, players should work towards preventing hunger and ensuring adequate hydration leading up to their matches. Players should aim to consume a meal comprising 1 to 2 g·kg^−^^1^ of body weight of carbohydrate, three to four hours before the match and top up glycogen and energy levels with low–moderate GI snacks one to two hours prior to a match. Food choices should be familiar, and low in fat and spice to minimize interference with digestion (e.g., banana, oat-based cereal bars). Players should follow up with the appropriate recovery methods suggested below. 

During competition, players may have to play multiple matches in a day and compete over several consecutive days. Players competing in multiple matches in a single day should ingest carbohydrate drinks (e.g., commercial sports drinks) or gels during game play to maintain motor skill proficiency and mitigate the cognitive function decline resulting from fatigue [87]. The aim is to maintain sufficient glycogen stores for ongoing and subsequent matches. While the ingestion of carbohydrate generally improved mental performance, there appears to be an inverted-U dose–response relationship between carbohydrate intake and performance, with an optimal dose of 25 g [88]. This suggests that table tennis players should not consume more than 350 mL of a standard carbohydrate–electrolyte drink (7% carbohydrate) in one go (e.g., during a match interval or time outs) and should instead sip on the drinks. For back-to-back matches, players should continue to sip on carbohydrate drinks and gels to maintain body glycogen stores. 

The current world top player for women’s singles, Chen Meng, revealed in a post-match interview after a grueling semi-final at a Grand Smash event (equivalent to a Grand Slam event for Tennis) that a tight best-of-seven-games match was very physically demanding, and that she experienced “low blood sugar” at one point during her match. She managed this by snacking regularly during breaks, highlighting the importance of nutrition during competition.

#### 3.3.3. Nutritional Strategies for Recovery 

The aim of recovery nutrition is to replace nutrients and fluids lost during exercise and to optimize the physiological readiness of the body prior to the next exercise bout. The timing and composition of the recovery meal or snack is largely dependent on the duration and intensity of the exercise and when the next exercise bout will occur [9]. The key areas of nutritional recovery involve restoring muscle and liver glycogen stores, the replacement of fluid and electrolytes lost in sweat, promoting protein synthesis for muscle repair and adaptation, and mitigating the negative effects of exercise such as immune disturbance and inflammatory responses [47,89]. 

Players who undergo training twice a day and wish to recover from the first training session should aim to consume a meal or snack immediately post exercise. Players should aim to consume carbohydrate at 1.0 to 1.2 g per kilogram of body weight at frequent intervals for up to 4 h post exercise and include protein-rich foods that provide 0.3 g·kg^−1^ of body weight of quality protein [9]. To rehydrate optimally, players should aim to ingest fluid together with meals or foods containing sodium for optimal absorption [47]. For refueling between matches on multi-match day, players should consume carbohydrate snacks or drinks that are moderate to high in GI for speedy glycogen replenishment, especially for matches that are less than 4 h apart.

For overnight recovery, players can also consider having a protein-rich meal or snack of about 25 to 40 g total protein prior to bed to maximize overnight protein synthesis and recovery [47,90]. Players recovering from single session training or light training can consume their usual meal within 60 min after exercise. Their recovery meal should provide carbohydrate, quality protein at 0.3 to 4 g·kg^−1^ of body weight, and fruits and vegetables for micronutrients and antioxidants.

### 3.4. Limitations

The majority of table-tennis-related research was conducted among players from Europe and North and South America. As such, available data may not be representative of Asian players. Another limitation of this review is that only English language studies were included for the analysis. Data from articles in other languages such as Chinese, Japanese and Korean were excluded from this review. Finally, as the sport is still growing and developing, there is a general paucity of nutrition research in this sport. 

## 4. Conclusions 

Based on the current understanding of the demands of table tennis, nutrition has potential in optimizing performance. Sport nutrition professionals should work closely with athletes and coaches to ensure that athletes consume the right amounts and types of nutrients for training and competition. 

Future nutrition research on table tennis can consider the following:

- Identifying physiological and strength tests that can more accurately depict the requirements of table tennis game play.
- Investigating the ideal body composition variation limits for training and competition.
- Profile nutritional adequacy and quality of elite table tennis players.
- Prevalence of vitamin D insufficiency or deficiency among table tennis players.
- Potential of lutein and zeaxanthin in improving table tennis performance.
- Profile lactic acid production in table tennis game play (post ball material change).

## Data Availability

No new data were created or analyzed in this study. Data sharing is not applicable to this article.
